# Epidemiology of hepatitis B virus infection in people living in poverty in the central-west region of Brazil

**DOI:** 10.1186/s12889-019-6828-8

**Published:** 2019-04-29

**Authors:** Lara Cristina da Cunha Guimarães, Sandra Brunini, Rafael Alves Guimarães, Hélio Galdino-Júnior, Ruth Minamisava, Vanessa Elias da Cunha, Jordana Rúbia Souza Santos, Elisângela de Paula Silveira- Lacerda, Christiane Moreira Souza, Vera Lúcia Brandão de Oliveira, Gabriela Cavalcante Albernaz, Thiago Guida de Menezes, Giovanni Rezza

**Affiliations:** 1 Faculty of Nursing, Federal University of Goiás, Goiânia, Goiás, Brazil; 2 Instituto de Patologia Tropical e Saúde Pública, Federal University of Goiás, Goiânia, Goiás, Brazil; 3 Instituto de Ciências Biológicas, Federal University of Goiás, Goiânia, Goiás, Brazil; 4 Laboratório de Análises Clínicas e Estudo em Saúde (LACES), Federal University of Goiás, Goiânia, Goiás, Brazil; 5 Secretaria de Estado da Saúde de Goiás/Coordenação Estadual de Controle das Hepatites Virais – CECHV, Goiânia, Goiás, Brazil; 6 Department of Infectious Diseases, Istituto Superiore di Sanitá, Rome, Italy

**Keywords:** Hepatitis B infection, Poverty, Epidemiology, immunisation

## Abstract

**Background:**

People living in poverty (PLP) are highly vulnerable to hepatitis B virus (HBV) infection. This study aimed to investigate the epidemiology of HBV infection in PLP in the metropolitan region of Goiânia, Goiás State, in the Central-West Region of Brazil.

**Methods:**

A cross-sectional study was conducted from August to December 2016 in adults aged ≥12 years living in poverty. The following serological markers for HBV were investigated: hepatitis B surface antigen (HBsAg), antibody to HBV core antigen (total anti-HBc), IgM anti-HBc, and hepatitis B surface antibody (anti-HBs), which were detected by enzyme-linked immunosorbent assay (ELISA). Poisson regression analysis with robust variance was performed to verify the factors associated with HBV exposure.

**Results:**

The study included 378 participants. The overall prevalence rate of HBV (any viral marker) was 9.8% (95% confidence interval [CI], 7.2–13.2). The prevalence rate of HBsAg in combination with total anti-HBc was 0.8% (95% CI, 0.3–2.4), total anti-HBc in combination with anti-HBs was 7.7% (95% CI, 5.4–10.9), and total anti-HBc alone was 1.3% (95% CI, 0.5–3.0) in the population. Furthermore, isolated positivity for anti-HBs was identified in only 25.4% (95% CI, 21.3–30.0) of the participants. Multiple regression analysis revealed that age (adjusted prevalence ratio [APR], 1.04; 95% CI, 1.01–1.07), female sex (APR, 2.18; 95% CI, 1.01–4.73), sexual intercourse under the influence of alcohol (APR, 2.49; 95% CI, 1.36–7.06), and exposure to *Treponema pallidum* (APR, 3.10; 95% CI, 1.36–7.06) were associated with HBV exposure.

**Conclusion:**

There was a high prevalence of HBV exposure in PLP in the Central-West Region of Brazil, indicating significant viral spread of the infection. Additionally, there was low serological evidence of immunisation against hepatitis B, indicating that a large proportion of the participants in this study are susceptible to the infection. The results support the need for public health policies that facilitate access to the existing healthcare services in hard-to-reach groups with special regard to immunisation programmes against hepatitis B.

## Background

Hepatitis B virus (HBV) infection is considered a serious public health problem worldwide, especially in less developed countries. It is estimated that 70% of new chronic HBV infections occur in low-income countries [1]. It is also estimated that 257 million people worldwide are chronic carriers of HBV and that there were 887,220 deaths as a result of the infection in 2015, most of which are related to complications such as cirrhosis and hepatocellular carcinoma [2].

Although Brazil has a low prevalence of HBV infection, it has the second largest population of individuals with positive hepatitis B surface antigen (HBsAg), accounting for 16.7% of all infections from the American Region [3]. In 2010, a population-based study conducted in adults aged 20–69 years in all macro-regions of Brazil estimated the prevalence of HBV exposure (11.6% positivity for total antibody to HBV core antigen [anti-HBc]) and infection (0.6% positivity for HBsAg). The lowest and highest prevalence of exposure were found in the Southeast (7.9%) and North (14.7%) macro-regions, respectively [4].

In a modelling study, performed with a Delphi process that included a literature review of PubMed and Embase, followed by interviews with experts, it was found that the prevalence rate of HBV infection (HBsAg positive) in the population of Brazil was 0.6%, where only 28% individuals were diagnosed (212,000) and only 12% of the population was treated. In children up to 5 years of age, the prevalence rate of infection is < 0.1%, which may be related to the high rates of vaccination coverage; 86% of children living in Brazil complete the vaccination schedule before reaching 1 year of age [5].

HBV can be transmitted by parenteral, sexual, vertical, and horizontal routes. Thus, individuals who engage in risky behaviours, such as the use of psychoactive substances, unprotected sex, multiple sexual partners, early initiation of sexual activity, and sharing of personal objects, are more vulnerable to HBV infection [6]. In addition to behavioural risk factors, social determinants such as low income and low level of schooling may contribute to increased exposure to HBV [6, 7].

People living in poverty (PLP) are significantly vulnerable to infectious diseases in general, and hepatitis B in particular, due to poor living conditions and/or difficulties in accessing healthcare. The concentration of poverty is associated with scarcity of basic services [8], resources [9], education [10], and employment opportunities, resulting in deterioration of quality of life of the residents [11, 12]. Moreover, the absence of primary healthcare services makes it difficult to access preventive services [13]. Lack of educational opportunities may limit the access to sexually transmitted infection (STI) prevention courses that are generally offered in schools. Furthermore, economic factors such as unemployment and poverty can promote risky behaviours such as inconsistent condom use and multiple sexual partnerships [14, 15], thus increasing an individual’s risk of acquiring an STI, such as hepatitis B [8].

In 2016, Brazil was ranked 96 in Healthcare Access and Quality Index by the Global Burden of Disease [16], setting up a country with great social inequalities and with a high number of PLP. According to the Brazilian Institute of Geography and Statistics (in Portuguese Instituto Brasileiro de Geografia e Estatística – IBGE) data, 16.27 million people were living in poverty in 2010, representing 8.5% of the country’s total population. Of these, 53.3% lived in urban areas, where the majority of the Brazilian population resides (84.4%) [17]. In Latin America, Brazil is one of the countries with the highest prevalence of HBV associated with the lower socioeconomic class [18]. Investigations among PLP have shown variations in the prevalence rate of HBV exposure in Brazil, ranging from 5.9% in adolescents in Goiânia (Goiás State, Central-West Region) [19] to 40.7% in the population of Buriticupu (Maranhão State, Northeast Region) [20].

However, studies that address exposure to HBV and factors associated with HBV in the general PLP population are still lacking, especially in the Central-West Region of Brazil. Most of the studies published to date address specific population groups (waste collectors, homeless, and immigrants) or do not present analytical approaches to the determinants associated with HBV. Thus, there is a gap in the literature on the epidemiology of hepatitis B in PLP using home-based and population-based surveys. This study intended to contribute to the monitoring of the prevalence of hepatitis B and to the understanding of the exposure profile and associated risk factors in Brazilian PLP. This is necessary to implement actions, services, and policies that meet the specific need of this population. The present study aimed to investigate the epidemiology of hepatitis B infection in PLP of the metropolitan region of Goiânia, Goiás State, Central-West Region of Brazil.

## Methods

### Design, population, and study sites

This is a cross-sectional study conducted among PLP in the metropolitan region of Goiânia, the capital of Goiás State, Central-West Region of Brazil, which has a population of 1,466,105 inhabitants.

**Fig. 1:**
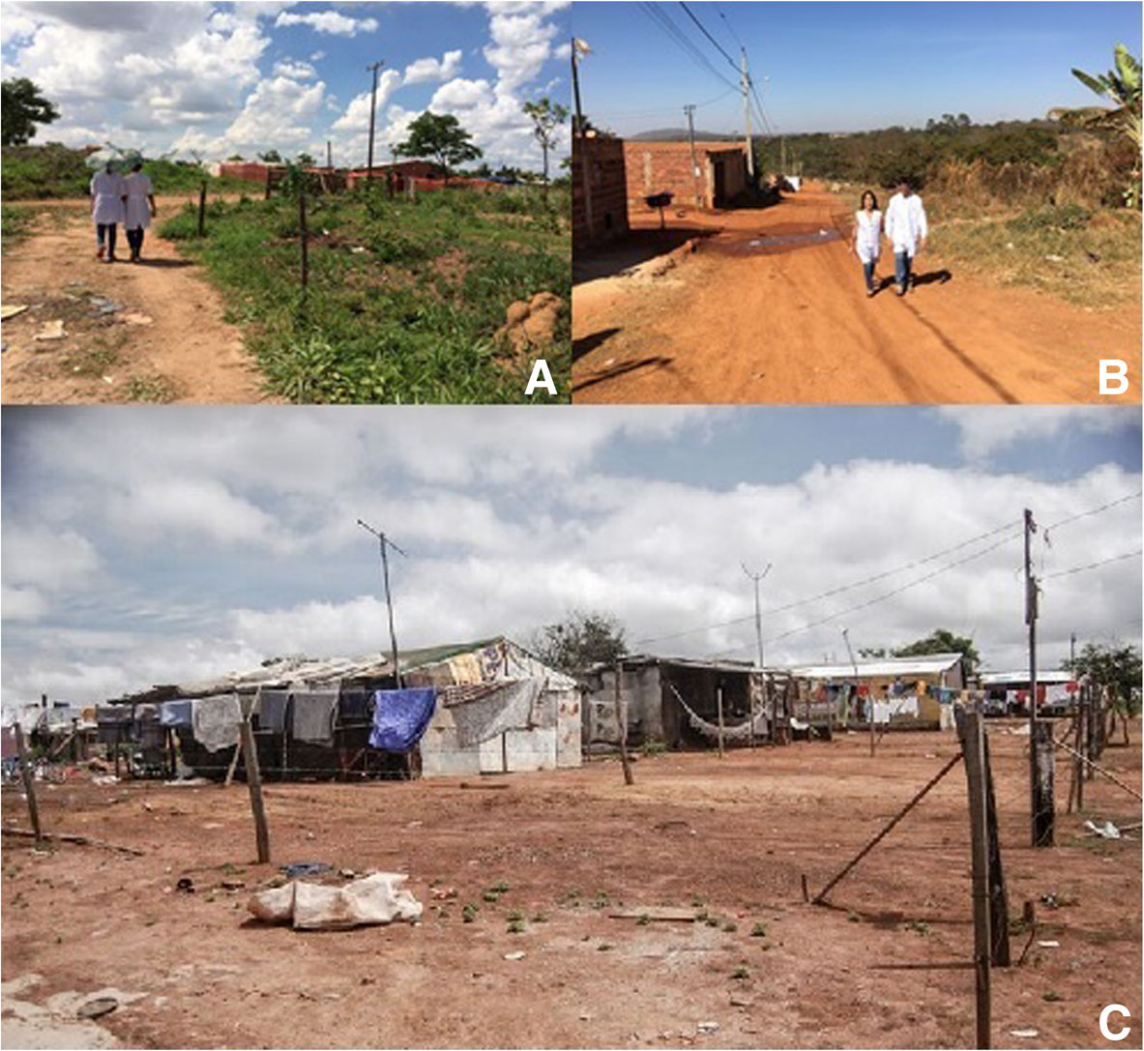
Areas of study sites. **a**–**b**, Neighbourhood 1; **c**, Neighbourhood 2

In the metropolitan region of Goiânia, there is an area where, until 2013, all the wastes generated in Goiânia were destined and accumulated without any separation of common or infectious wastes. Over the years, a population group settled in favelas in the adjacent areas, living in precarious conditions and working with the collection of these wastes. Since the closure of the zone and the creation of the sanitary landfill, some families received masonry housing from the government and were transferred to a new living space (Neighbourhood 1, Fig. 1 a, b). Other families remained in the area (Neighbourhood 2, Fig. 1 c). Still, families who moved to neighbourhood 1, despite living in masonry homes and geographically closer to public health and education facilities, remained without access to basic sanitation and paved streets where sewage runs in the open. Therefore, these two neighbourhoods were selected in this study because they reflect signs of extreme poverty within the metropolitan region and have in common, beyond the social precariousness, the history of cohabitation with the waste.

According to Kageyama and Hoffman [21], income is an unreliable indicator of living conditions that characterise poverty due to inaccurate information provided by the people. Thus, it is necessary to resort to variables that are related to consumption expenditures as a more advantageous and specific method of identifying PLP [21].

In the neighbourhoods, there are a large number of canvas, cardboard, or wood residences without access to pipe-borne water, basic sanitation, and public lighting. There are no pavements in the streets. There are no family healthcare units or other healthcare services for the population. Under this scenario, reports of exploitation and child labour, abuse and domestic violence against women, the use of illicit drugs, and sex workers are frequent.

In this context, the criteria of the Brazilian Economic Classification (in Portuguese *Critério da Classificação Econômica do Brasil* – CCEB) were adopted for the selection of the participants for the present study [22, 23]. The CCEB is a system of proxy punctuation of the consumption capacity of a household, expressing the wealth of the residences and the purchasing power of the families. In this investigation, the CCEB 2015 version was used. This takes into account the possessions and quantity of household comfort goods (presence of automobiles, microcomputer, and digital versatile disc player, degree of schooling of the head of the family (illiterate to complete higher education), and access to public services (pipe-borne water and paved streets), which together represent a permanent family income construct [22, 23]. For each CCEB variable, a specific score ranging from 0 to 100 is assigned, where the higher the score, the higher the socioeconomic classification of the family. The CCEB system generates a score that stratifies families into the following socioeconomic classes: A (scores from 45 to 100), B1 (38–44), B2 (29–37), C1 (23–28), C2 (17–22), and D–E (0–16) [23]. Families who belong to D–E classification are families living in poverty.

In the present study, people aged 12 years or older who, according to the CCEB, belonged to classes D–E, that is, who had a score in the CCEB equal to or less than 16 points, were included. Participants with apparent cognitive impairment or major incapacitating psychic disorders were considered ineligible.

The sample size was calculated for the finite population, and a statistical power of 80% (β = 20%) with a significance level of 95% (α = 0.05) and a global prevalence rate of HBV of 12.8%, according to Marinho et al. [24], with design effect correction of 2.0 were considered. Therefore, the minimum sample size needed to estimate the prevalence rate in the study population was 325 participants, which was increased by 10% to correct for loss and refusal, totalling a sample of 358 participants. This study included 378 participants.

### Data collection

Data collection occurred between August and December 2016, and the recruitment occurred through home visits. All the houses in both neighbourhoods were visited. For each eligible household, a participant was drawn from the list of residents aged ≥12 years. Next, the selected resident was invited to participate in the study. In case of the absence of the resident at the time of the interview, at least three attempts to contact residents in each sampled home were made at different periods [25, 26]. There was no substitution in the event of an absence of the resident at the end of the third visit.

The data were collected through face-to-face interviews in the households, using a structured instrument to obtain socio-demographic data and other potential factors associated with HBV infection. After the interview, rapid testing for syphilis was performed locally, and 5 ml of blood was collected for serological tests for hepatitis B.

### Laboratory analysis

HBV-related markers (HBsAg, total anti-HBc, IgM anti-HBc, and hepatitis B surface antibody [anti-HBs]) were detected by enzyme-linked immunosorbent assay (ELISA) according to each protocol. The results were delivered to the study participants during post-test counselling.

### Study variables

#### Dependent variable

The presence or combination of the following HBV exposure markers was considered to be the endpoint variable: (i) HBsAg and total anti-HBc, (ii) total anti-HBc and anti-HBs, or (iii) total anti-HBc isolated.

#### Independent variables

The following independent variables were analysed:

(i) Sociodemographic: age (years), gender (male or female) as gender proxy, schooling (full years of formal study), and marital status (married, separated/divorced/widower, or single).

(ii) Non-sexual risk factors: tattoo (no or yes), body piercing (no or yes), sharing of personal objects (toothbrushes, pliers, razor, pliers, or nail clippers) (no or yes), history of hepatitis in the family (no or yes), history of incarceration (no or yes), marijuana use (no or yes), use of intranasal cocaine (no or yes), crack use (no or yes), injectable drug use (no or yes), alcohol use (no or yes), and alcohol dependence (no or yes).

Alcohol dependence was assessed using the CAGE (an acronym for the following: ‘Cut down, Annoyedby criticism, Guilty, and Eye-opener’) questionnaire [27], which includes these four questions: (1) Have you ever felt you needed to cut down on your drinking? (2) Have people annoyed you by criticising your drinking? (3) Have you ever felt guilty about drinking? (4) Have you ever felt you needed a drink first thing in the morning (eye-opener) to steady your nerves or to get rid of a hangover? This questionnaire was used with a cut-off of two affirmative responses suggesting positive screening for alcohol abuse or dependence [27, 28]. In Brazil, CAGE assessment was validated in 1983 by Masur and Monteiro, who found a sensitivity of 88% and a specificity of 83% for the detection of alcohol dependence [29].

(iii) Sexual risk factors: use of condoms during sexual intercourse (always or never/sometimes), condom use at the last sexual intercourse (no or yes), practice of anal sex (no or yes), homosexual relationship (no or yes), sexual relations with sex workers (no or yes), sexual intercourse with illicit drug users (no or yes), sexual intercourse with STI carriers (no or yes), sexual intercourse under illicit drug use (no or yes), sexual intercourse under the influence of alcohol (always or never/sometimes), number of sexual partners in life, number of sexual partners last year, age of first sexual intercourse, STI history (no or yes), and exposure to *Treponema pallidum* (negative or positive).

The exposure to *Treponema pallidum* (*T. pallidum*), the etiologic agent of syphilis, was evaluated by the rapid test, performed after the interview.

### Statistical analysis

The data were analysed using the Stata software, version 14.0 [30]. Initially, the Anderson-Darling (AD) test was performed to verify the normality of the quantitative variables [31]. Three levels of analysis were then conducted: (i) descriptive, (ii) bivariate regression, and (iii) multiple regression.

In the descriptive analysis, quantitative variables were presented as median and interquartile range (IQR) due to the absence of normality in the AD test and qualitative variables in absolute and relative frequencies. The prevalence of HBV serological markers was estimated with 95% confidence interval (CI).

Subsequently, bivariate Poisson analysis was performed to verify the potential factors associated with HBV exposure. Variables that were included in the Poisson regression model were those with robust variance [14, 32, 33] with *p*-value < 0.20 in the bivariate analysis. The final modelling of factors associated with HBV infection was adjusted for age, sex, education, history of hepatitis in the family, injecting drug use, sexual intercourse under the influence of alcohol, the age at the time of sexual intercourse, STI history, and exposure to *T. pallidum*. Variables with *p*-value < 0.05 in multivariate analysis were considered to be statistically significant.

### Ethical aspects

This study was approved by the Ethics Committee of the Hospital das Clínicas of the Federal University of Goiás, with protocol number 1.669.956/2016. Informed written consent was obtained from all participants. For minors, consent was also obtained from their legal guardians. Individuals with positive test results were referred to specialist services for clinical evaluation and treatment.

## Results

Between August and December 2016, 455 households were visited. Of these, 13 residences were excluded from the study because none of their residents were found even after three visits on different days and times. In five households, all residents refused to participate in the survey. Thus, the study applied the CCEB questionnaire in 437 residences. Of these, 33 residents who did not meet the poverty criteria were excluded from the study; thus, overall data from 404 residences were included.

Thus, only one resident was randomly selected from all residents in the eligible households (*N* = 404) and invited to participate in the study. Of these, 26 refused to participate in the study, resulting in a study population of 378 participants.

The socio-demographic and behavioural characteristics of the participants are shown in Table 1. The median age was 31 (IQR, 21–42) years, and for schooling was 7 (IQR, 5–9) years. The majority of participants were married (59.3%).

**Table 1:** Sociodemographic characteristics and potential risk factors for hepatitis B among the study population (Goiânia, Goiás, Central-West Region of Brazil, 2016)

Variables	*N* = 378	%
Age (years), median (IQR)	31.0 (21.0–42.0)	
Education (years), median (IQR)	7.0 (5.0–9.0)	
Sex		
Male	188	49.7
Female	190	50.3
Marital status		
Married	224	59.3
Divorced/separated/widowed	32	8.5
Single	122	32.3
Sharing of personal objects^a^		
No	113	30.0
Yes	264	70.0
Tattoo		
No	257	68.0
Yes	121	32.0
*Body piercing*		
No	427	88.9
Yes	41	11.1
History of hepatitis in the family		
No	281	87.8
Yes	39	12.2
History of imprisonment^b^		
No	290	81.0
Yes	68	19.0
Injecting drug use^b^		
No	374	98.9
Yes	4	1.1
Alcohol use^c^		
No	191	50.5
Yes	187	49.5
Alcoholic dependence		
No	246	69.3
Yes	109	30.7
Marijuana use^c^		
No	286	75.7
Yes	92	24.3
Intranasal cocaine use^c^		
No	331	87.6
Yes	47	12.4
Crack use^c^		
No	347	91.8
Yes	32	8.2
STIs history^d^		
No	337	89.2
Yes	41	10.8
*Treponema pallidum* exposure		
Negative	342	90.5
Positive	36	9.5
Condom use^a^		
Never	65	18.8
Sometimes/always	280	81.2
Condom use at last sexual intercourse		
No	261	76.1
Yes	82	23.9
Anal sex^a^		
No	215	63.8
Yes	122	36.2
Sexual relation homosexual^a^		
No	316	92.4
Yes	26	7.6
Sexual intercourse with illicit drug users^a^		
No	171	49.6
Yes	174	50.4
Sexual intercourse with sex workers^a^		
No	265	77.3
Yes	78	22.7
Sexual intercourse with STIs carriers^a^		
No	313	91.3
Yes	30	8.7
Sexual intercourse under the influence of alcohol^a^		
Never	201	58.3
Sometimes/always	144	41.7
Age of first sexual intercourse, median (IQR)	15.0 (13.0–17.0)	
Number of sexual partners^b^, median (IQR)	5.0 (3.0–15.0)	
Number of sexual partners^a^, median (IQR)	1.0 (1.0–1.0)	

Abbreviations: *IQR* Interquartile range

^a^Previous 6 months

^b^Lifetime

^c^Pevious month

^d^Pevious 12 months

The main known risk factors for HBV infection found in our study were the following: sharing of personal objects (70.0%), tattoo (32.0%), body piercing (11.1%), imprisonment (19.0%), alcohol use (49.5%), alcohol dependence (30.7%), marijuana use (24.3%), intranasal cocaine use (12.4%) and crack use (8.2%), STI history (10.8%), inconsistent condom use (81.2%), sexual intercourse under the influence of alcohol (41.7%), and exposure to *T. pallidum* (9.5%). The median age at first sexual intercourse and the number of lifetime sexual partners were 15 years and five partners, respectively.

Markers of exposure to HBV were detected in 40 participants, representing an overall prevalence rate of 9.8% (95% CI, 7.2–13.2). Three participants (0.8%) tested positive for HBsAg, 29 for anti-HBc and anti-HBs (7.7%), and five for isolated anti-HBc (1.3%). Isolated positivity for anti-HBs was observed in 25.4% (95% CI, 21.3–30.0) of the participants, suggesting that they were vaccinated against HBV. All of the participants with total anti-HBc positivity were tested for IgM anti-HBc, but none of them tested positive. Finally, the majority of the studied population (64.8%) were considered to be susceptible to HBV infection (Table 2).

**Table 2:** Prevalence of serological markers of hepatitis B virus in people living in poverty. Goiânia, Goiás, Central-West Region of Brazil, 2016

Serological markers	N = 378	%	95.0% CI
HbsAg + Total anti-HBc	3	0.8	0.3–2.3
Total Anti-HBc + anti-HBs	29	7.7	5.4–10.8
Total Anti-HBc isolated	5	1.3	0.5–3.0
IgM Anti-HBc	0	0	0
Some exposure markers	37	9.8	7.2–13.2
Anti-Hbs isolated	96	25.4	21.3–30.0
Absence of marker (susceptible)	245	64.8	59.9–69.5

Abbreviations: HbsAg: Hepatitis B surface antigen; Total anti-HBc: antibody to HBV core antigen; anti-HBs: hepatitis B surface antibody; IgM Anti-HBc: IgM antibody to hepatitis B core antigen; 95.0% CI: 95.0% Confidence Interval

In the Poisson bivariate analysis, exposure to HBV was associated with higher age, history of hepatitis in the family, injecting drug use, alcohol use, exposure to *T. pallidum*, sexual intercourse under the influence of alcohol, and age at first sexual intercourse *(p*-value < 0.05). These variables and sex (*p*-value = 0.112) were included in the Poisson regression model with robust variance (Table 3).

**Table 3:** Bivariate analysis of the potential factors associated with HBV exposure in people living in poverty. Goiânia, Goiás, Central-West Region of Brazil, 2016

Variables	Total^a^	HBV exposure				PR (95.0% CI)	*p*-value^b^
		Negative		Positive			
Age (years)	33.0 (23.0–46)	32.0 (21.0–42.5)		47.0 (34.5–54.0)		1.03 (1.01–1.06)	<0.001
Education (years)	7.0 (4.0–9.0)	7.0 (4.0–9.0)		7.0 (2.0–10.0)		0.97 (0.89–1.07)	0.657
Sex							
Male	144	130	90.3	14	9.7	1.00	
Female	138	115	83.3	23	16.7	1.71 (0.88–3.33)	0.112
Marital status							
Married	167	147	88.0	20	12.0	1.00	
Divorced/separated/widowed	28	23	82.1	5	17.9	1.49 (0.55–3.97)	0.424
Single	87	75	86.2	12	13.8	1.15 (0.56–2.35)	0.699
Tattoo							
No	195	171	87.7	23	12.3	1,00	
Yes	87	74	85.1	13	14.9	1.21 (0.61–2.38)	0.573
Body piercing							
No	219	187	85.4	32	14.6	1,00	
Yes	63	58	92.1	5	7.9	0.54 (0.21–1.39)	0.204
Sharing of personal objects							
No	83	73	88.0	10	12.0	1,00	
Yes	198	172	86.9	26	13.1	1.08 (0.52–2.26)	0.817
History of hepatitis in the family							
No	204	178	87.3	26	12.7	1,00	
Yes	32	23	71.9	9	28.1	2.20 (1.03–4.70)	0.041
Blood transfusion							
No	245	216	88.2	29	11.8	1,00	
Yes	30	24	80.0	6	20.0	1.68 (0.70–4.06)	0.242
History of imprisonment							
No	220	192	87.3	28	12.7	1,00	
Yes	50	42	84.0	8	16.0	1,11 (0,54-2,26)	0,772
Marijuana use							
No	212	182	85.8	30	14.2	1,00	
Yes	70	63	90.0	7	10.0	0.70 (0.31–1.60)	0.408
Crack use							
No	253	221	87.4	32	12.6	1,00	
Yes	29	24	82.8	5	17.2	1.36 (0.53–3.49)	0.519
Intranasal cocaine use							
No	244	211	86.5	33	13.5	1,00	
Yes	38	34	89.5	4	10.5	0.77 (0.27–2.19)	0.636
Injecting drug use							
No	278	243	87.4	35	12.6	1,00	
Yes	4	2	50.0	2	50.0	3.97 (0.95–16.51)	0.058
Alcohol use							
No	139	128	92.1	11	7.9	1,00	
Yes	143	117	81.8	26	18.2	2.29 (1.13–4.64)	0.021
Alcoholic dependence							
No	181	160	88.4	21	11.6	1,00	
Yes	84	69	82.1	15	17.9	1.53 (0.79–2.98)	0.202
STIs history							
No	249	219	88.0	30	12.0	1,00	
Yes	33	26	78.8	7	21.2	1.76 (0.77–4.00)	0.178
*Treponema* pallidum exposure							
Negative	252	226	89.7	26	10.3	1,00	
Positive	30	19	63.3	11	36.7	3.55 (1.75–7.19)	<0.001
Condom use at last sexual intercourse							
Yes	60	52	86.7	8	13.3	1,00	
No	195	166	85.1	29	14.9	1.11 (0.50–2.43)	0.785
Anal sex							
No	153	129	84.3	24	15.7	1,00	
Yes	98	85	86.7	13	13.3	0.84 (0.43–1.66)	0.626
Sexual relation homosexual							
No	233	201	86.3	32	13.7	1,00	
Yes	21	16	76.2	5	23.8	1.73 (0.67–4.44)	0.253
Sexual intercourse with illicit drug users							
No	230	197	85.7	33	14.3	1,00	
Yes	26	22	84.6	4	15.4	0.86 (0.45–1.64)	0.653
Sexual intercourse with sex workers							
No	195	166	85.1	29	14.9	1,00	
Yes	60	52	86.7	8	13.3	00.89 (0.40–1.96)	0.785
Sexual intercourse with STIs carriers							
No	230	196	85.2	34	14.8	1,00	
Yes	25	22	88.0	3	12.0	0.81 (0.24–2.64)	0.729
Sexual intercourse under the influence of alcohol							
Never	139	126	90.6	13	9.4	1,00	
Sometimes/always	117	117	79.5	24	20.5	1.19 (1.11–4.30)	0.027
Age of first sexual intercourse	15.0 (13.5–17.0)	15.0 (14.0–16.0)		15.0 (13.0–17.5)		1.04 (0.99–1.11)	0.100
Number of sexual partners	6.0 (3.0–15.0)	6.0 (3.0–15.0)		9.0 (2.2–28.7)		1.00 (0.99–1.00)	0.792
Number of sexual partners	1.0 (1.0–1.0)	1.0 (1.0–1.0)		1.0 (1.0–1.0)		1.02 (0.90–1.15)	0.716

Abbreviations: HBV: hepatitis B virus; PR: Prevalence ratio; STIs: Sexually transmitted infections; 95.0% CI: 95.0% Confidence interval

Note: quantitative variables presented as medians and IQR

^a^Number of valid answers

^b^Wald statistic

Table 4 shows the multiple regression analysis of factors associated with HBV exposure. After adjusting for the model, age (adjusted prevalence ratio [APR], 1.04; 95% CI, 1.01–1.07), female sex (APR, 2.18; 95% CI, 1.01–4.73), sexual intercourse under the influence of alcohol (APR, 2.49; 95% CI, 1.09–5.67), and *T. pallidum* exposure (APR, 3.10; 95% CI, 1.36–7.06) remained as the independent risk factors associated with HBV exposure (Table 4).

**Table 4:** Multiple regression analysis of risk factors associated with HBV exposure in individuals living in poverty in the metropolitan region of Goiânia, Central Brazil, 2016

Risk factors	APR (95.0% CI)^a^	*p*-value^b^
Age (years)	1.04 (1.01–1.07)	0.002
Education (years)	1.06 (0.96–1.17)	0.243
Female sex	2.18 (1.01–4.73)	0.048
History of hepatitis in the family	1.66 (0.73–3.77)	0.219
Injecting drug use	1.61 (0.29–8.78)	0.578
Sexual intercourse under the influence of alcohol	2.49 (1.09–5.67)	0.029
Age of first sexual intercourse	1.04 (0.98–1.10)	0.168
STIs history	0.64 (0.21–1.95)	0.439
*Treponema pallidum* exposure	3.10 (1.36–7.06)	0.007

Abbreviations: HBV: hepatitis B virus; APR: Adjusted prevalence ratio; STIs: Sexually transmitted infections; 95.0% CI: 95.0% Confidence interval

^a^Model adjusted for age, sex, education, history of hepatitis in the family, injecting drug use, sexual intercourse under the influence of alcohol, age at first sexual intercourse, STIs history and exposure to *Treponema pallidum*; Wald chi-squared test; McFadden’s *R*^2^: 0.154

^b^Wald statistic

## Discussion

This study investigated the prevalence and risk factors of HBV infection in PLP in the Central-West Region of Brazil. The research findings provide important data on the epidemiology of HBV infection, which may intensify the prevention and control actions in this population. The observed prevalence was similar to that found in the general population. However, approximately 3/5 of the population showed absence of any marker of infection, indicating viral susceptibility. Age, female sex, sexual intercourse under the influence of alcohol, and *T. pallidum* exposure were the independent risk factors associated with HBV exposure.

In the present study, the prevalence rate of HBV exposure was 9.8% (95% CI, 7.2–13.2%), similar to that found in the general population aged 20–69 years in Brazil (11.6%; 95% CI, 10.7–12.4%) and the Central-West Region (12.7%; 95% CI, 10.9–14.5%) [4]. In Brazil, few population-based studies investigated the epidemiology of hepatitis B in PLP [34]. Considering the studies conducted in other PLP or proxy populations, the results were similar to low-income adolescents (5.9%; 95% CI, 4.2–8.0%) [19] and recyclable material pickers (12.8%; 95% CI, 9.8–16.2%) [35] of Goiânia, Central-West Region. These results indicated that the Central-West Region has a low endemicity for hepatitis B. On the other hand, the results of this investigation were lower than those estimated in recyclable waste pickers in the City of Santos (São Paulo State, South-eastern Brazil) (34.4%; 95% CI, 28.5–40.2%) [24] and in the low-income population of Buriticupu (Maranhão State, Northeast Region) (40.7; 95% CI, 34.7–47.0%) [20]. In addition to the methodological differences and variations in risk behaviours, the first study included an older population than that of the present study. Moreover, in the case of the second study, the North Region had an intermediate endemicity when compared to the Central-West Region. These factors may explain the differences of prevalence found between these studies.

The vaccine card is considered to be the gold standard for the evaluation of vaccination coverage. However, when unavailable, isolated detection of the anti-HBs marker can be used as an indicator of the vaccine status of populations. In this study, only 25.4% of the participants presented a serological profile consistent with previous immunisation, and 64.8% did not present hepatitis B serologic markers, indicating viral susceptibility. Low serological evidence of HBV immunisation has also been found in other studies in PLP or in those with extreme social vulnerability in Brazil [20, 35, 36].

The Global Health Sector Strategy on Viral Hepatitis by the World Health Organization (WHO) proposed to eliminate hepatitis B by 2030 treating 80% of patients with HBV and HCV and achieving 90% vaccination coverage of the three doses in childhood by 2020. Although several member states of the WHO, including Brazil, are working on strategies to control hepatitis and eliminate the epidemic, the challenges are still immense, especially when it comes to financial issues [37]. Globally, only 11% of HBV and HCV cases are diagnosed. There is a dire need to hasten hepatitis diagnosis and search the missing millions living with hepatitis who remain undiagnosed [38]. The diagnosis may be even more underestimated in PLP, because of inaccessibility to healthcare services.

Currently, in Brazil, hepatitis B vaccine is widely available at most public health network to the populace regardless of age and/or level of vulnerability [39]. However, the low immunisation rate and the high susceptibility rate shown in this study indicate that extremely poor populations have difficulties or fail to access the immunisation programme. Consequently, this will increase the susceptibility to more advanced age groups, which could constitute clusters of maintenance of the virus transmission chain. This is often related to the absence of primary healthcare services in impoverished regions. The presence of susceptible population groups may negatively impact the extent of elimination of hepatitis B transmission by 2030.

Multiple regression analysis, as well as the other studies, revealed an association between exposure to HBV and age increase [14, 20, 36, 40]. The displacement of the epidemiological curve of HBV in populations with higher age groups can be explained by the Brazilian policy of increasing the age for eligibility of hepatitis B vaccine over the years [34]. This result supports the importance of offering and intensifying vaccination against hepatitis B in elderly [36]. The association between age and HBV exposure may also reflect the cumulative risk of HBV infection associated with lifelong sexual and parenteral exposure [14, 41].

Alcohol consumption has a complex association with health status, and the determination of the damages caused by it is even more complicated because it is influenced by the volume and consumption pattern of the individual. In 2016, 60 to 79.9% of Brazilian men and 40 to 59.9% of Brazilian women reported alcohol consumption in the last 12 months, with a daily intake of 3 to 4 doses and 1 to 2 doses, respectively, accounting for a total of 47.3 deaths per 100,000 inhabitants [42]. Alcohol consumption during intercourse was also associated with exposure to HBV, which is a well-documented association in the national and international literature [34, 43]. Alcohol consumption during intercourse decreases the perception of risk and increases the chance of risky behaviours, such as inconsistent condom uses and multiple sexual partners. These behaviours potentiate the risk for HBV infection [43, 44].

In the present study, there was an association between exposure to *T. pallidum*, the etiological agent of syphilis, and exposure to HBV. Of the total number of participants exposed to HBV, 29.7% were also exposed to *T. pallidum*. The presence of ulcerative lesions caused by syphilis significantly increases the risk of infection and transmission of HBV and other STI such as human immunodeficiency virus (HIV) [45, 46]. This result highlights the need for testing for multiple STI, such as hepatitis B and C, syphilis, and HIV, in PLP.

This study had some limitations. The cross-sectional nature of this study does not allow the establishment of causality between HBV exposure and predictor variables. The frequencies of risk behaviours may have been underestimated due to recall bias and socially desirable responses to particular issues, such as number of partners, use of condoms, and consumption of licit and illicit substances. The generalisability of our findings is limited because our study included PLP in only one Brazilian region. However, this study represented one of the first investigations on the epidemiology of HBV infection, which may contribute to the implementation of control policies in this population.

## Conclusion

In conclusion, there was a high prevalence of HBV exposure in PLP in the Central-West Region of Brazil, indicating significant viral spread of the infection. High rates of risk behaviour were found, suggesting that this population is at a high risk of acquiring HBV and other STI. Moreover, there was low serological evidence of immunisation against hepatitis B, indicating that a large proportion of the participants in this study are susceptible to this infection. The results support the need for public health policies to promote the access of hard-to-reach groups by the existing healthcare services, especially the immunisation programme against hepatitis B.

## Abbreviations

95% CI: 95% confidence interval
AD: Anderson-Darling
Anti-HBc total: Total antibody to HBV core antigen
Anti-HBs: Hepatitis B surface antibody
APR: Adjusted prevalence ratio
HBsAg: Surface antigen of the hepatitis B virus
HBV: Hepatitis B virus
IgM anti-HBc: IgM antibody to hepatitis B core antigen
IIQ: Interquartile range
PLP: People living in poverty
PR: Prevalence ratio
